# Front-line Bevacizumab in combination with Oxaliplatin, Leucovorin and 5-Fluorouracil (FOLFOX) in patients with metastatic colorectal cancer: a multicenter phase II study

**DOI:** 10.1186/1471-2407-7-91

**Published:** 2007-05-30

**Authors:** Christos Emmanouilides, Georgia Sfakiotaki, Nikolaos Androulakis, Kostas Kalbakis, Charalambos Christophylakis, Antonia Kalykaki, Lambros Vamvakas, Athanasios Kotsakis, Sofia Agelaki, Eleni Diamandidou, Nikolaos Touroutoglou, Adam Chatzidakis, Vassilis Georgoulias, Dimitris Mavroudis, John Souglakos

**Affiliations:** 1 Department of Medical Oncology, Interbalkan Medical Center, Thessaloniki, Greece; 2 Department of Medical Oncology, University General Hospital of Heraklion, Heraklion, Greece; 3 First Department of Medical Oncology, IASO General Hospital, Athens, Greece; 4 Department of Radiology, University General Hospital of Heraklion, Heraklion, Greece

## Abstract

**Purpose:**

To evaluate the efficacy and the toxicity of front line FOLFOX4 combined with bevacizumab in patients with metastatsic CRC (mCRC).

**Patients and Methods:**

Chemotherapy-naïve patients with mCRC, received bevacizumab (5 mg/kg every 2 weeks d_1_), oxaliplatin (85 mg/m^2 ^on d_1_), leucovorin (200 mg/m^2^) on days 1 and 2 and 5-Fluorouracil (400 mg/m^2 ^as i.v. bolus and 600 mg/m^2 ^as 22 h i.v. continuous infusion on days 1 and 2) every 2 weeks.

**Results:**

Fifty three patients (46 with a PS 0–1) were enrolled. Complete and partial response was achieved in eight (15.1%) and 28 (52.8%) patients, respectively (ORR: 67.9%; 95% C.I.: 53.8%–92%); 11 (20.7%) patients had stable disease and six (11.3%) progressive disease. With a median follow up period of 13.5 months, time to tumor progression was 11 months while the median survival has not yet been reached; the probability of 1-, 2- and 3- year survival was 79.8%, 63.8% and 58.3%, respectively; Two patients relapsed during the follow up period. Eight (15%) patients underwent metastasectomy with R0 resections. Grade 3–4 neutropenia occurred in 15.1% of patients and one (1.9%) of them presented febrile neutropenia. Non-hematologic toxicity included grade 3 diarrhea (7.6%) and grade 2 and 3 neurotoxicity in 16.9 and 15.1% of patients, respectively. One (1.9%) patient presented pulmonary embolism and one (1.9%) cardiac ischaemia. There was one (1.9%) sudden death after the first cycle.

**Conclusion:**

The combination of FOLFOX4/bevacizumab appears to be highly effective, well tolerated and merits further evaluation in patients with mCRC.

## Background

Colorectal cancer (CRC) is a major cause of cancer morbidity and mortality worldwide [1] accounting for 8% of all malignant tumors in adults [2]. Despite that macroscopically curative surgical resection is possible in 70–80% of patients at diagnosis, almost half of them will develop local or/and metastatic recurrence and will die of the disease.

Although, historically, chemotherapy was used for palliation of symptoms, during the last few years the median overall survival of patients with advanced CRC has been substantially increased from 12 months to about 21–22 months when all of the available chemotherapeutic agents are administered [3]. Therefore treatment of metastatic colorectal cancer (mCRC) has changed considerably in the recent years. Combinations of 5-fluorouracil/Leucovorin (5-FU/LV) containing both bolus (Roswell Park) and infusional administration (De Gramont schedule) combined with a second active drug, either irinotecan [4] or oxaliplatin are accepted as the mainstay of first-line treatment, while the choice of a particular drug to combine with 5FU does not influence overall survival[5].

The advent of targeted therapy further expanded treatment options for patients with mCRC. In particular, inhibition of angiogenesis by blocking Vascular Endothelial Growth Factor (VEGF) using the monoclonal antibody bevacizumab led to further improvement in the outcome of patients with mCRC. Indeed, randomized studies demonstrated that the addition of bevacizumab to either 5FU/LV [6-8], or to an Irinotecan-5FU/LV combination (IFL) [9] as first-line treatment of mCRC was associated with improved objective response rate, time to tumor progression and overall survival.

During the last years, the IFL regimen (weekly irinotecan and IV push administration of 5FU and LV) no longer represents the gold standard of front line treatment of mCRC and it was replaced by the combinations of irinotecan or oxaliplatin with the infusional 5-FU regimens (FOLFIRI and FOLFOX, respectively) [10,11]. A recent study (E3200) [12] demonstrated that the addition of bevacizumab improved the activity of second-line oxaliplatin-containing combination in patients with mCRC. However in this study the effect of the combination on survival and response rate was modest, reflecting the more advanced stage of the disease in such patients.

Since there was no information concerning the efficacy and tolerance of the combination of FOLFOX4 plus bevacizumab as front line treatment of patients with mCRC, the Gastrointestinal (GI) Working Group of the Hellenic Oncology Research Group (HORG) decided to conduct this multicenter phase II study.

## Methods

### Eligibility criteria

Chemotherapy naïve patients, aged ≥ 18 years with histologically documented mCRC were enrolled; other eligibility criteria included: patients who had received prior adjuvant 5-FU-based chemotherapy were eligible if they had remained free of disease for at least 6 months after the completion of adjuvant therapy; performance status (ECOG) 0–2; at least one bidimensionally measurable lesion of ≥ 2 cm; a life expectancy of at least 3 months; adequate hematologic parameters (absolute neutrophil count ≥ 1.5 × 10^9^/L and platelets ≥ 100 × 10^9^/L); creatinine and total bilirubin ≤ 1.25 times the upper limit of normal; aspartate and alanine aminotransferases ≤ 3.0 times the upper limit of normal; absence of active infection or malnutrition (loss of more than 10% of body weight); absence of a second primary tumor other than non-melanoma skin cancer or *in situ *cervical carcinoma; patients receiving palliative radiotherapy had to have measurable metastatic disease outside the irradiation fields.

Patients with operable metastatic disease, clinically significant cardiovascular disease or major surgery within one month prior to study registration were excluded from the study. Other exclusion criteria included: pregnancy or lactation, regular use of aspirin (more than 325 mg per day) or other nonsteroidal anti-inflammatory agents; preexisting bleeding diatheses or coagulopathy or the need for full-dose anticoagulation; history of deep vein thrombosis within one year prior to registration; uncontrolled hypertension; pre-treatment proteinuria grade 2 or more and known central nervous system metastases. Patients with chronic diarrhea, or prior irradiation affecting more than 30% of the active bone marrow were also excluded. The study was approved by the Ethics and Scientific Committees of each participating center. All patients gave written informed consent in order to participate in the study.

### Chemotherapy

Bevacizumab (Avastin^®^; Roche, Bale, Suisse) was given prior to chemotherapy at a dose of 5 mg/kg every 2 weeks, as a 2-hours infusion intravenous (IV) during the first cycle, as a 1-hour IV infusion during the second cycle and as a 30 min IV infusion in the subsequent cycles; L-OHP (Eloxatin^®^; Sanofi-Aventis, Collegeville, USA) was administered on day 1 at the dose of 85 mg/m^2 ^as a 2-hours IV infusion; LV was given at the dose of 200 mg/m^2 ^as a 2-hour IV infusion, followed by 5-FU 400 mg/m^2 ^as IV bolus, and then, 600 mg/m^2 ^as a 22-hour continuous IV infusion, on days 1 and 2. Routine antiemetic prophylaxis with a 5-hydroxytryptamine-3-receptor antagonist was used in both study groups. Treatment was administered every 2 weeks until disease progression or unacceptable toxicity, or until the patient declined further treatment. No maintenance therapy with Bevacizumab after chemotherapy discontinuation was planned.

Patients were assessed for toxicity before each cycle using the National Cancer Institute Common Toxicity Criteria [13]. Chemotherapy was delayed until recovery if neutrophils were less than 1.5 × 10^9^/L or platelets less than 100 × 10^9^/L or for significant persisting nonhematologic toxicity. Doses of LOHP and 5-FU were reduced by 15% in subsequent cycles in case of grade 4 neutropenia or grade 3–4 thrombocytopenia lasting for more than 3 days or in case of febrile neutropenia. No prophylactic administration of granulocyte colony-stimulating factor (G-CSF) was allowed. G-CSF was used for the treatment of febrile neutropenia. Doses of 5-FU was reduced by 15% in subsequent cycles in case of grade 2–4 diarrhea, stomatitis or dermatitis. L-OHP dose was reduced by 15% in cases of persistent (≥ 14 days) paresthesia or temporary (7 to 14 days) painful paresthesia or functional impairment. In cases of persistent (≥ 14 days) painful paresthesia or functional impairment, L-OHP was omitted in subsequent cycles until recovery. Bevacizumab was permanently discontinued in patients developing gastrointestinal perforation, wound dehiscence requiring medical intervention, serious bleeding, nephrotic syndrome, or hypertensive crisis. Temporary discontinuation of Bevacizumab administration was implemented in patients with evidence of moderate to severe proteinuria and in patients with severe hypertension that was not controlled with medical management. Doses of bevacizumab and chemotherapy were recalculated if the patient's weight changed by more than 10 percent during the study.

### Patients' evaluation

Pre-treatment evaluation included a detailed medical history and physical examination, a complete blood cell count (CBC) with differential and platelet count, blood chemistry, urine analysis, serum levels of carcinoembryonic antigen (CEA) and computed tomography scans (CT) of the chest and abdomen. Pretreatment evaluation had to be performed within 2 weeks prior to study entry. During treatment, a CBC with differential and platelets count was performed weekly and in case of grade 3–4 neutropenia, thrombocytopenia or febrile neutropenia it was performed daily until hematologic recovery. In addition, patients were clinically assessed and blood chemistry and urine analysis were performed before each treatment cycle. Response to treatment was evaluated every 2 months (4 chemotherapy cycles) or sooner if clinically indicated. After the treatment period the patients without disease progression, were evaluated with clinical examination and CT scans every 2 months.

The Response Evaluation Criteria in Solid Tumors (RECIST) were used to assess tumour responses [14]. Complete response was defined as disappearance of all disease without the appearance of new lesions. Partial response was defined as a reduction of at least 30% in the sum of the products of the longest diameters of all measurable lesions. Disease progression was defined as an increase of at least 20% in measurable tumor or an unequivocal increase in the size of non-measurable lesions or the appearance of new lesions. After partial response, tumor measurements exceeding 20% of the maximal extent of a previously observed reduction constituted progression. Patients who did not meet the definitions of response or progression were classified as having stable disease. All objective responses were confirmed by a follow-up CT scan at least 4 weeks following documentation of the response.

### Statistical Considerations

The primary end point of the study was the tumor response rate. The study followed the optimal Simon two-step design. If a minimum objective response rate exceeding 45% was observed in the first 16 patients, 35 additional patients had to be enrolled (α = 0.05, power 80%). Secondary objectives were the tolerance of the regimen as well as response duration, time to tumor progression and overall survival time. The duration of response was defined as the time from the first documentation of response to disease progression. The time to tumor progression (TTP) was determined by the interval between the initiation of treatment and the date of first documentation of disease progression or the date of death from any cause. Overall survival (OS) was defined as the time from treatment initiation to death. The follow up time was defined as the period from treatment initiation to the study's cutoff date (for alive patients). TTP and OS were estimated by the Kaplan-Meier method [15], and the confidence intervals for response rates were calculated using methods for exact binomial confidence intervals [16].

## Results

### Patients' characteristics

Between May 2004 and September 2005, 53 patients were enrolled, at three institutions and all of them received at least one cycle of chemotherapy. Most of the patients (median age 65 years) were males (56.6%) with good performance status (ECOG 0–1: 86.7%) (Table 1). Fourteen (26.4%) patients were newly diagnosed with metastatic disease whereas 39 patients (73.6%) had prior resection of the primary tumor. Of the latter group 13 had prior adjuvant chemotherapy and six (11.3%) had also received radiation therapy. The median time elapsed between the diagnosis of metastasis and study entry was 1 month (range, 0–2.5 months), while the median time between initial disease diagnosis and the first metastasis was 16.5 months (range, 7–68 months), for patients with initially operable disease. Thirty-four (64%) patients presented liver metastasis and the median number of metastatic sites was 2/patient (range, 2–5). Overall, approximately 22.6% of the patients were classified as high risk according to the Kohne prognostic index [17].

### Compliance with the treatment

Five hundred and thirty nine chemotherapy courses were administered (median, 12 courses/patient; range, 2–20). Fifty (9.3%) chemotherapy courses were delayed for a median of 7 days (range, 1–10) due to hematological (n = 21; 3.9%), non-hematologic (n = 10; 1.8%) toxicity, and for reasons unrelated to disease or treatment (n = 19; 3.6%). The median interval between cycles was 16 days (range, 14–24). Dose reduction of cytotoxic agents was required in 27 (5%) cycles because of hematologic (n = 11; 2.1%), and non-hematologic (n = 16; 2.9%) toxicity. The delivered relative dose intensity was 92% and 94% of the protocol planned doses for L-OHP and 5-FU/LV, respectively. At the time of this analysis, 49 (92.4%) patients have discontinued treatment for the following reasons: progressive disease (n = 40 patients), unacceptable neurotoxicity (n = 6 patients), death without disease progression (n = 3 patients).

### Treatment efficacy

**Figure 1:**
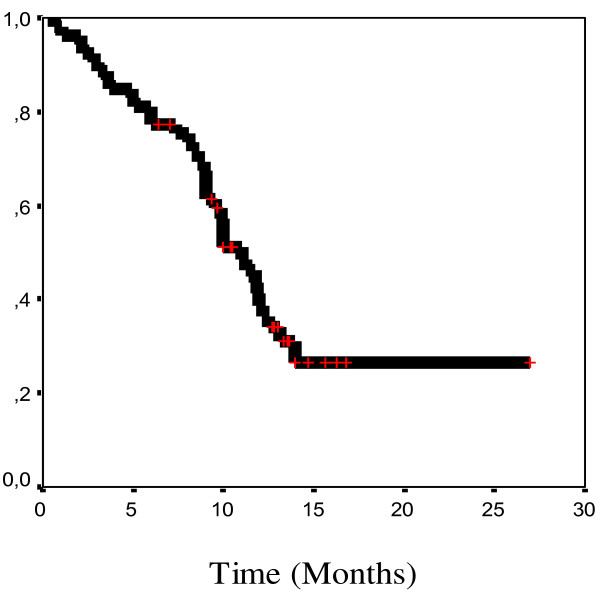
Kaplan-Meier time to tumor progression (TTP) of patients treated with FOLFOX/Bevacizumab.

In an intention-to-treat-analysis eight (15.1%) patients achieved a documented complete response (CR) and 28 (52.8%) a partial response (PR), for an overall response rate (ORR) of 67.9% (95% C.I.: 53.8%–92%). All objective response were documented with a following CT scan at least 4 weeks later. In addition, 11 (20.8%) patients had stable disease (SD) and six (11.4%) progressive disease (PD). The median response duration was 8 months (range, 3–21.2) and the median time to initial documentation of response was 2 months (range, 2–4). The median TTP was 11 months (range, 0.6–28) (Figure 1). After a median follow-up period of 20 months (range, 0.6–38), 33 (71.7%) patients were still alive. The median overall survival time has not yet been reached (range 0.6–38.0); the probability of 1-, 2- and 3- year's survival was 79.8%, 63.8%, and 58.3 respectively.

In eight (15.1%) patients with initially unresectable metastatic lesions in the liver (n = 6) or the lungs (n = 2), a metastasectomy was eventually performed following the study treatment. The administration of bevacizumab was interrupted 4 weeks prior to planned surgery. R0 resection could be achieved in all patients (Table 2) and all patients were uneventfully recovered. Two of those patients have relapsed 11 and 13 months post-metastasectomy; after a median follow up of 10 months (range, 4–18 months), all metastasectomized patients were alive at the time of analysis.

### Toxicity

Neutropenia and neuropathy were the most common toxic effects of the combination. (Table 3). Grade 3–4 neutropenia was observed in eight (15.1%) patients and one (1.9%) of them developed febrile neutropenia requiring hospitalization and treatment with IV antibiotics. Grade 3 anemia and thrombocytopenia occurred in one (1.9%) patient each. Grade 3 diarrhea developed in four (7.6%) patients. Neurosensory toxicity was observed in 35 (66%) patients and reached grade 3 in eight (15.1%) of them. Cold-induced dysesthesia was reported in 18 (33.9%) patients. Paresthesia without pain occurred in nine (16.9%) patients. Cumulative paresthesia occurred in eight (15.1%) patients but without functional impairment. The estimated incidence of grade 2 and 3 neurotoxicity, attributed to L-OHP exposure, was 9% and 32% after 6 cycles, respectively. Other grade 3 or 4 toxicities were infrequent. One patient died of sudden death, because of cardiac rhythm abnormalities, after the first chemotherapy cycle. Another patient developed unstable angina pectoris without infraction which was attributed to 5-FU-induced coronary angiospasm. A third patient presented an episode of pulmonary embolism but he was recovered uneventfully. Only one patient presented severe gastrointestinal blending from the primary tumor in the descending colon (Table 4). In total four treatment-related admissions to the hospital were reported. The death rate during the first 60 days of treatment was 3.8% (95% CI, 1.0%–5.3%).

## Discussion

The present study reports the efficacy and safety results of the bevacizumab-FOLFOX4 combination as first-line treatment for patients with mCRC. Although bevacizumab has been accepted by most clinicians as a component of the first-line treatment of mCRC, there is insufficient information concerning the outcome of patients using this combination in the first line setting.

The current multicenter phase II trial demonstrated that the three-drug combination of bevacizumab, L-OHP and 5-FU/LV produced a high response rate (67.9%) with a median duration of response of 7 months and a median TTP of 11 months. The projected probabilities of 1- (79.8%), 2- (63.8%) and 3- (58.3 %) year survival rates were very promising. In addition, eight (15.1%) patients with prior unresectable liver or pulmonary metastatic lesions underwent secondary metastasectomies and R0 resection was able to be performed in all of them. These efficacy results are among the highest reported in the literature for any chemotherapy regimen in mCRC. It is noteworthy that these results were obtained with acceptable toxicity.

Although, the present study has the limitations of any phase II study, the patient population was representative for this particular disease; indeed, almost one quarter of the patients were classified as high risk, and more than half of them as intermediate risk according to the Kohne score, indicating the poor prognosis of those patients.

It is well known that bevacizumab enhances the activity of the co-administered chemotherapy regimens that have been so far studied as first or second line treatment of colorectal cancer [6,7,9,18-21]. This has been documented by prospective randomized studies in the first-line setting, using either 5-FU/LV, or IFL regimen [7,9,20,21]; in the second line-setting, it enhances the activity of the FOLFOX4 regimen, in bevacizumab-naïve patients according to the E3200 study [12]. In the latter study, the effect on survival and objective response rate was rather modest, reflecting the more advanced stage of the disease in such patients. Thus, the observations regarding efficacy and possibly toxicity are not readily transferable when the same drug combination is used as first line treatment. This clearly necessitated the study of the same combination in the first line. In the same E3200 study, although the toxicity of the chemotherapy combination was not enhanced by the addition of bevacizumab, four cases of bowel perforation were observed. Such concerns further enhance the importance of a meticulous analysis of the activity and the toxicity of the combination in the first line setting. A recent preliminary report of a randomized phase II trial suggested that the addition of bevacizumab improves the response rate, time to treatment failure and median overall survival when it was added to standard L-OHP-based chemotherapy [22]. The only phase III (NO 16966) reported until now, demonstrated a relatively small but statistically significant (1.5 months, p = 0.0023) improvement for progression free survival when bevacizumab was added to L-OHP based regimens XELOX or FOLFOX4, although that survival data were missing [23].

Since the benefit of bevacizumab seems to be independent of the combination used as front line treatment, it is not clear whether the clinical benefit of bevacizumab is specific to the combination of irinotecan and 5-FU/LV used in the phase III study. In fact, combining FOLFOX-4 with bevacizumab as second line treatment and at higher doses than those used in the pivotal study, increased the response and the overall survival rates albeit to a smaller extend. In addition, non-randomized studies suggest an advantage to combining Bevacizumab with oxaliplatin plus bolus 5FU in the first line setting [22].

In summary, our results indicate that the combination of FOLFOX and bevacizumab is feasible and highly effective, and merits further evaluation in a phase III trial.

## List of Abbreviations

FOLFOX: Folinic acid, Fluorouracil, Oxaliplatin

CRC: colorectal cancer

mCRC: colorectal cancer

LV: Leucovorin

5-FU: 5-Fluorouracil

VEGF: Vascular Endothelial Growth Factor

HORG: Hellenic Oncology Research Group

ECOG: Eastern Oncology Group

L-OHP: Oxaliplatin

CBC: complete blood cell count

CEA: carcinoembryonic antigen

CT: computed tomography

RECIST: Response Evaluation Criteria in Solid Tumors

CR: complete response

PR: partial response

RR: response rate

SD: Stable disease

PD: Progressive disease

TTP: time to tumor progression

OS: Overall survival

## Competing interests

The author(s) declare that they have no competing interests.

## Aurthors' contributions

CE has made substantial contribution to conception and design, interpretation of data; has been involved in drafting the manuscript

GS has made substantial contribution to conception and design, acquisition of data; interpretation of data;

NA has made substantial contribution acquisition of data; interpretation of data;

KK has made substantial contribution in interpretation of data

CC carried out the statistical analyis

AK has made substantial contribution acquisition of data; interpretation of data;

LV has made substantial contribution in acquisition of data

All authors read and approved the final manuscript

## Pre-publication history

The pre-publication history for this paper can be accessed here:



## Figures and Tables

**Figure 2:**
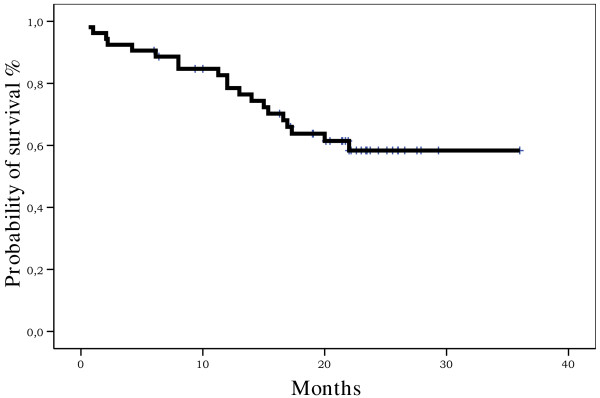
Kaplan-Meier overall survival (OS) of patients treated with FOLFOX/Bevacizumab.

**Table 1:** Patients' characteristics

	**No of patients**	**%**
**Number of patients enrolled**	**53**	
**Age (years)**		
**Median (range)**	65 (18 – 78)	
**Sex**		
Male	30	56.6
Female	23	43.4
**Performance status (WHO)**		
0	26	49
1	20	37.7
2	7	13.2
**Tumor Origin**		
Colon	36	67.9
Rectum	17	32.1
**Prior Treatment**		
Surgery	39	73.6
Adjuvant chemotherapy	13	24.5
Adjuvant radiotherapy	6	11.3
**Abnormal ALP^# ^level at baseline**	21	39.6
**Abnormal WBC^ level at baseline**	10	18.9
Abnormal PLT* level at baseline	6	11.3
**Kohne prognostic index**		
Low risk	11	20.7
Intermediate risk	30	56.6
High risk	12	22.6
**Sites of disease**		
Local Abdominal Mass	11	20.7
Liver	34	64.2
Lung	18	34
Lymph Nodes	17	32
**Number of metastatic sites**		
1	14	26.4
2	30	56.6
≥ 3 median	9	17
Median (range)	2 (1–5)	

^#^ALP: Alkaline Phosphatase, ^WBC: White cells Blood Count, *PLT: Platelets

**Table 2:** Characteristics of patients who underwent secondary metastasectomy

	**Metastatic sites**	**Response**	**TTP (months)**	**Relapse**
**Patient 1**	Liver	CR	12	No
**Patient 2**	Liver	PR	13	Yes (Liver)
**Patient 3**	Lung	PR	4	No
**Patient 4**	Liver	PR	9	No
**Patient 5**	Liver	CR	11	Yes (Lung)
**Patient 6**	Lung	PR	18	No
**Patient 7**	Liver	PR	7	No
**Patient 8**	Liver	PR	14	No

**Table 3:** Worst toxicity grade by patient during all cycles of treatment with the FOLFOX/BEVACIZUMAB combination

	**Grade**							
	
	**1**		**2**		**3**		**4**	
	**n**	**%**	**n**	**%**	**n**	**%**	**n**	**%**
Neutropenia	4	7.5	10	18.9	6	11.3	2	3.8
Febrile neutropenia	-	-	-	-	1	1.9	-	-
Anemia	39	73.6	5	9.4	1	1.9	-	-
Thrombocytopenia	8	15.1	2	3.8	1	1.9	-	-
Nausea	6	11.3	1	1.9	1	1.9	-	-
Vomiting	4	7.5	2	3.8	-	-	-	-
Diarrhea	10	18.9	7	13.2	4	7.6	-	-
Mucocitis	3	5.7	-	-	-	-	-	-
Constipation	9	17.0	3	5.7	-	-	-	-
Neurotoxicity	18	33.9	9	16.9	8	15.1	-	-
Alopecia	12	22.6	2	3.8	-	-	-	-
Fatigue	13	24.5	6	11.3	-	-	-	-

**Table 4:** Bevacizumab possibly related adverse events

	**Grade**							
	
	**1**		**2**		**3**		**4**	
	**n**	**%**	**n**	**%**	**n**	**%**	**n**	**%**
Pulmonary embolism	-	-	-	-	-	-	1	1.9
Cardiac ischaemia	-	-	-	-	1	1.9	-	-
Epistaxis	4	7.6	1	1.9				
Gastrointestinal bleeding	-	-	-	-	1	1.9	-	-
Hypertension	5	9.5	2	3.8	-	-	-	
Proteinuria	7	13.2	4	7.6	-	-	-	-
